# Hexaaqua­cobalt(II) bis­{[*N*-(4-meth­oxy-2-oxidobenzyl­idene)glycyl­glycinato]copper(II)} hexa­hydrate

**DOI:** 10.1107/S1600536809040045

**Published:** 2009-10-07

**Authors:** Gan-Bing Yao, Jia-Xun Jiang, Li-Min Yuan, Xiao-Ming Ren

**Affiliations:** a College of Chemistry and Chemical Engineering, Yangzhou University, Yangzhou 225002, People’s Republic of China; b Key Laboratory of Environmental Material and Environmental Engineering of Jiangsu Province, Yangzhou 225002, People’s Republic of China

## Abstract

In the crystal structure of the title compound, [Co(H_2_O)_6_][Cu(C_12_H_11_N_2_O_5_)]_2_·6H_2_O, the Co^II^ atom is located on an inversion center and coordinated by six water mol­ecules in a slightly distorted octa­hedral geometry. The Cu^II^ atom is chelated by the Schiff base ligand in a distorted CuN_2_O_2_ square-planar geometry. An extensive O—H⋯O hydrogen-bonding network is present in the crystal structure.

## Related literature

For the magnetic properties of Schiff base complexes, see: Ion *et al.* (2009 ▶); Wu *et al.* (2007 ▶); Costes *et al.* (2006 ▶) and for their optical properties, see: Akine *et al.* (2008 ▶). 
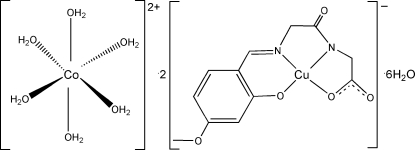


## Experimental

### 

#### Crystal data

[Co(H_2_O)_6_][Cu(C_12_H_11_N_2_O_5_)]_2_·6H_2_O
*M* *_r_* = 928.66
Triclinic, 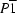

*a* = 7.834 (2) Å
*b* = 10.835 (3) Å
*c* = 11.474 (3) Å
α = 76.705 (4)°
β = 76.616 (5)°
γ = 81.085 (4)°
*V* = 916.7 (4) Å^3^
*Z* = 1
Mo *K*α radiation
μ = 1.69 mm^−1^
*T* = 296 K
0.35 × 0.30 × 0.25 mm

#### Data collection

Bruker SMART APEX CCD diffractometer
Absorption correction: multi-scan (*SADABS*; Bruker, 2001 ▶) *T*_min_ = 0.561, *T*_max_ = 0.658
4773 measured reflections
3345 independent reflections
2801 reflections with *I* > 2σ(*I*)
*R*_int_ = 0.072

#### Refinement

*R*[*F* ^2^ > 2σ(*F* ^2^)] = 0.042
*wR*(*F* ^2^) = 0.105
*S* = 1.01
3345 reflections
242 parameters
H-atom parameters constrained
Δρ_max_ = 0.59 e Å^−3^
Δρ_min_ = −0.73 e Å^−3^

### 

Data collection: *SMART* (Bruker, 2002 ▶); cell refinement: *SAINT-Plus* (Bruker, 2003 ▶); data reduction: *SAINT-Plus*; program(s) used to solve structure: *SHELXTL* (Sheldrick, 2008 ▶); program(s) used to refine structure: *SHELXTL*; molecular graphics: *SHELXTL*; software used to prepare material for publication: *SHELXTL*.

## Supplementary Material

Crystal structure: contains datablocks I, global. DOI: 10.1107/S1600536809040045/xu2621sup1.cif

Structure factors: contains datablocks I. DOI: 10.1107/S1600536809040045/xu2621Isup2.hkl

Additional supplementary materials:  crystallographic information; 3D view; checkCIF report

## Figures and Tables

**Table 1:** Hydrogen-bond geometry (Å, °)

*D*—H⋯*A*	*D*—H	H⋯*A*	*D*⋯*A*	*D*—H⋯*A*
O6—H6*A*⋯O11	0.83	1.94	2.771 (4)	174
O6—H6*B*⋯O3^i^	0.84	1.93	2.765 (3)	169
O7—H7*A*⋯O9^ii^	0.83	1.95	2.757 (3)	165
O7—H7*B*⋯O10	0.81	1.94	2.723 (3)	162
O8—H8*C*⋯O2^iii^	0.85	2.31	2.812 (3)	118
O9—H9*A*⋯O10	0.85	1.99	2.769 (4)	152
O9—H9*B*⋯O1	0.85	1.96	2.798 (3)	172
O10—H10*C*⋯O2^iv^	0.85	2.02	2.783 (3)	149
O10—H10*D*⋯O4^i^	0.85	2.00	2.844 (4)	173
O11—H11*A*⋯O2^v^	0.85	2.09	2.916 (3)	164
O11—H11*B*⋯O9	0.85	2.00	2.844 (4)	176

Symmetry codes: (i) 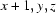; (ii) 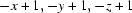; (iii) 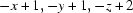; (iv) 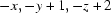; (v) 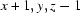.

**Fig. 1.**
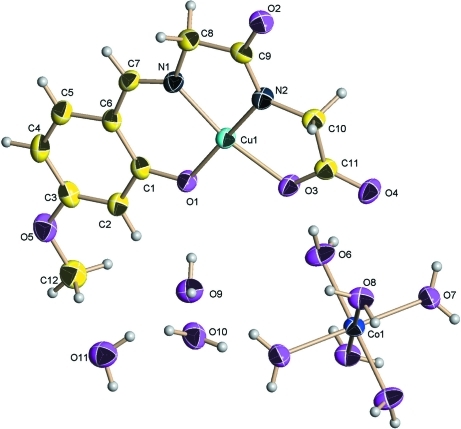
The molecular structure of (I), with atom labels and 50% probability displacement ellipsoids.

## supplementary crystallographic information

### Comment

In recent years, the design and synthesis of Schiff base complexes caused an increasing interest in coordination chemistry because they were potential optical, magnetic materials (Ion *et al.*, 2009; Wu *et al.*, 2007; Costes *et al.*, 2006; Akine *et al.*, 2008). Now, we present the synthesis and structure analysis of the title Schiff base complex derived from 4-methoxy-salicylaldehyde and glycylglycine.

The complex (I) crystallizes in the triclinic space group *P*1. The asymmetric unit consists of one [CuL]^-^ anion (*L* is a Schiff base derived from glycylglycine and 4-methoxy-salicylaldehyde), half Co(H_2_O)_6_^2+^ cation [Co(1), O(6), O(7), O(8)] and three uncoordinated aqua molecules [O(9), O(10), O(11)] in the complex (I) (Fig. 1). The deprotonated Schiff base is a triple negatively charged tetradentate chelate ligand, coordinating to the Cu(II) atom by one phenolate O atom [O(1)], one imine N atom [N(1)], one deprotonated amide N atom [N(2)] and one carboxylato O atom [O(3)]. [CuL]^-^ has approximately square-planar structure. The Cu(II) atom is in a slightly distorted square-planar environment with four donor atoms deviating from their mean plane by -0.0503 Å (N(1)), +0.0621 Å (N(2)), +0.0509 Å (O(1)) and -0.0494 Å (O(3)) (observed bond angles vary from 83.4 (1)° and 96.6 (1)°). The benzene ring [C(1)–C(6)] and the [O(1), C(1), C(6), C(7), N(1), Cu(1)] chelate ring are almost coplanar with a small dihedral angle of 0.1 (1)°, suggesting a large π-electron delocalization. The Co(II) atom lies on an inversion center and the coordination by six aqua ligands is slightly distorted octahedral. The six Co—O bonds in the structure are in the range of 2.075 (2) - 2.081 (2) Å. In the crystal structure, the [CuL]^-^ anions and [Co(H_2_O)_6_]^2+^ cations form well separated columns along the *a*-axis, which are further formed a three-dimensional network by hydrogen bonds (Table 1).

### Experimental

Glycylglycine (5 mmol), 4-methoxy-salicylaldehyde (5 mmol) and LiOH (10 mmol) were dissolved in MeOH/H_2_O (30 ml, v:v = 1:1) and refluxed for 30 min. Then Cu(ClO_4_)_2_.6H_2_O (5 mmol) was added to the solution and the resulting solution was adjusted to 9–11 by 5 M NaOH solution. After stirring at room temperature for 1 h, CoCl_2_.6H_2_O (2.5 mmol) was added. A violet precipitate was obtained immediately. After stirring for another 30 min and then filtrated, the precipitate was recrystallized from water. The violet crystals suitable for X-ray diffraction were obtained after one week (yield 30% based on Cu(ClO_4_)_2_.6H_2_O).

### Refinement

The water H atoms in (I) were located in a difference Fourier map and refined with a distance restraint of O—H = 0.83–0.85 Å and *U*_iso_(H) =1.5*U*_eq_(O). Other H atoms were positioned geometrically and constrained as riding atoms, with C—H distances of 0.93–0.97 Å and *U*_iso_(H) set to 1.2 or 1.5*U*_eq_(C) of the parent atom.

### Crystal data

**Table:**

[Co(H_2_O)_6_][Cu(C_12_H_11_N_2_O_5_)]_2_·6H_2_O	*Z* = 1
*M**_r_* = 928.66	*F*(000) = 479
Triclinic, *P*1	*D*_x_ = 1.682 Mg m^−^^3^
Hall symbol: -P 1	Mo *K*α radiation, λ = 0.71073 Å
*a* = 7.834 (2) Å	Cell parameters from 2213 reflections
*b* = 10.835 (3) Å	θ = 2.9–27.3°
*c* = 11.474 (3) Å	µ = 1.69 mm^−^^1^
α = 76.705 (4)°	*T* = 296 K
β = 76.616 (5)°	Block, violet
γ = 81.085 (4)°	0.35 × 0.30 × 0.25 mm
*V* = 916.7 (4) Å^3^	

### Data collection

**Table:**

Bruker SMART APEX CCD diffractometer	3345 independent reflections
Radiation source: fine-focus sealed tube	2801 reflections with *I* > 2σ(*I*)
graphite	*R*_int_ = 0.072
φ and ω scans	θ_max_ = 25.5°, θ_min_ = 1.9°
Absorption correction: multi-scan (*SADABS*; Bruker, 2001)	*h* = −9→9
*T*_min_ = 0.561, *T*_max_ = 0.658	*k* = −11→13
4773 measured reflections	*l* = −7→13

### Refinement

**Table:**

Refinement on *F*^2^	Primary atom site location: structure-invariant direct methods
Least-squares matrix: full	Secondary atom site location: difference Fourier map
*R*[*F*^2^ > 2σ(*F*^2^)] = 0.042	Hydrogen site location: inferred from neighbouring sites
*wR*(*F*^2^) = 0.105	H-atom parameters constrained
*S* = 1.01	*w* = 1/[σ^2^(*F*_o_^2^) + (0.0518*P*)^2^] where *P* = (*F*_o_^2^ + 2*F*_c_^2^)/3
3345 reflections	(Δ/σ)_max_ < 0.001
242 parameters	Δρ_max_ = 0.59 e Å^−^^3^
0 restraints	Δρ_min_ = −0.73 e Å^−^^3^

### Special details

**Table:**

Geometry. All e.s.d.'s (except the e.s.d. in the dihedral angle between two l.s. planes) are estimated using the full covariance matrix. The cell e.s.d.'s are taken into account individually in the estimation of e.s.d.'s in distances, angles and torsion angles; correlations between e.s.d.'s in cell parameters are only used when they are defined by crystal symmetry. An approximate (isotropic) treatment of cell e.s.d.'s is used for estimating e.s.d.'s involving l.s. planes.
Refinement. Refinement of *F*^2^ against ALL reflections. The weighted *R*-factor *wR* and goodness of fit *S* are based on *F*^2^, conventional *R*-factors *R* are based on *F*, with *F* set to zero for negative *F*^2^. The threshold expression of *F*^2^ > σ(*F*^2^) is used only for calculating *R*-factors(gt) *etc*. and is not relevant to the choice of reflections for refinement. *R*-factors based on *F*^2^ are statistically about twice as large as those based on *F*, and *R*- factors based on ALL data will be even larger.

### Fractional atomic coordinates and isotropic or equivalent isotropic displacement parameters (Å^2^)

**Table:**

	*x*	*y*	*z*	*U*_iso_*/*U*_eq_	
Cu1	0.03586 (5)	0.20388 (4)	0.98824 (3)	0.02809 (14)	
C1	0.2956 (4)	0.0148 (3)	0.9002 (3)	0.0288 (7)	
C2	0.4083 (4)	−0.0331 (3)	0.8024 (3)	0.0311 (7)	
H2	0.4107	0.0127	0.7229	0.037*	
C3	0.5146 (4)	−0.1458 (3)	0.8214 (3)	0.0341 (8)	
C4	0.5171 (5)	−0.2159 (3)	0.9394 (3)	0.0374 (8)	
H4	0.5912	−0.2913	0.9524	0.045*	
C5	0.4078 (5)	−0.1707 (3)	1.0351 (3)	0.0349 (8)	
H5	0.4084	−0.2174	1.1139	0.042*	
C6	0.2944 (4)	−0.0569 (3)	1.0203 (3)	0.0290 (7)	
C7	0.1895 (4)	−0.0188 (3)	1.1282 (3)	0.0315 (7)	
H7	0.2013	−0.0715	1.2029	0.038*	
C8	−0.0200 (4)	0.1156 (3)	1.2453 (3)	0.0355 (8)	
H8A	0.0605	0.1224	1.2957	0.043*	
H8B	−0.0942	0.0491	1.2897	0.043*	
C9	−0.1332 (4)	0.2406 (3)	1.2200 (3)	0.0300 (7)	
C10	−0.2110 (5)	0.4100 (3)	1.0531 (3)	0.0319 (8)	
H10A	−0.1755	0.4809	1.0770	0.038*	
H10B	−0.3368	0.4073	1.0840	0.038*	
C11	−0.1693 (4)	0.4271 (3)	0.9149 (3)	0.0311 (7)	
C12	0.6253 (5)	−0.1365 (4)	0.6071 (3)	0.0487 (10)	
H12A	0.5082	−0.1290	0.5921	0.073*	
H12B	0.7045	−0.1860	0.5535	0.073*	
H12C	0.6631	−0.0531	0.5921	0.073*	
N1	0.0808 (4)	0.0818 (3)	1.1303 (2)	0.0294 (6)	
N2	−0.1159 (4)	0.2923 (3)	1.1028 (2)	0.0302 (6)	
O1	0.1993 (3)	0.1241 (2)	0.87335 (19)	0.0314 (5)	
O2	−0.2285 (3)	0.2879 (2)	1.30684 (19)	0.0365 (6)	
O3	−0.0524 (3)	0.3465 (2)	0.86770 (18)	0.0337 (5)	
O4	−0.2476 (3)	0.5175 (2)	0.8547 (2)	0.0425 (6)	
O5	0.6258 (3)	−0.1980 (2)	0.7310 (2)	0.0436 (6)	
Co1	1.0000	0.5000	0.5000	0.02696 (17)	
O6	0.9561 (3)	0.3322 (2)	0.6289 (2)	0.0422 (6)	
H6A	0.8856	0.2887	0.6164	0.063*	
H6B	0.9396	0.3336	0.7039	0.063*	
O7	0.7348 (3)	0.5271 (2)	0.4914 (2)	0.0393 (6)	
H7A	0.7050	0.5904	0.4409	0.059*	
H7B	0.6609	0.5097	0.5534	0.059*	
O8	0.9612 (3)	0.6042 (2)	0.63661 (19)	0.0357 (6)	
H8D	0.9732	0.6820	0.6039	0.054*	
H8C	1.0367	0.5755	0.6815	0.054*	
O9	0.3966 (3)	0.2928 (2)	0.6876 (2)	0.0478 (7)	
H9A	0.4091	0.3533	0.7196	0.072*	
H9B	0.3299	0.2420	0.7389	0.072*	
O10	0.5032 (3)	0.5168 (3)	0.7098 (2)	0.0452 (6)	
H10C	0.4526	0.5916	0.6909	0.068*	
H10D	0.5707	0.5161	0.7582	0.068*	
O11	0.7326 (4)	0.1922 (3)	0.5697 (2)	0.0491 (7)	
H11A	0.7595	0.2283	0.4950	0.074*	
H11B	0.6310	0.2231	0.6018	0.074*	

### Atomic displacement parameters (Å^2^)

**Table:**

	*U*^11^	*U*^22^	*U*^33^	*U*^12^	*U*^13^	*U*^23^
Cu1	0.0315 (2)	0.0310 (2)	0.0209 (2)	0.00299 (17)	−0.00540 (16)	−0.00771 (16)
C1	0.0265 (16)	0.0292 (18)	0.0336 (18)	−0.0023 (14)	−0.0083 (14)	−0.0106 (14)
C2	0.0335 (18)	0.0302 (18)	0.0312 (18)	−0.0003 (14)	−0.0098 (14)	−0.0084 (14)
C3	0.0295 (17)	0.0325 (18)	0.046 (2)	−0.0015 (14)	−0.0095 (15)	−0.0177 (15)
C4	0.0353 (19)	0.0305 (19)	0.051 (2)	0.0035 (15)	−0.0192 (17)	−0.0109 (16)
C5	0.0366 (19)	0.0319 (19)	0.0382 (19)	−0.0023 (15)	−0.0156 (16)	−0.0042 (15)
C6	0.0278 (17)	0.0296 (18)	0.0322 (17)	−0.0018 (14)	−0.0112 (14)	−0.0072 (14)
C7	0.0341 (18)	0.0321 (19)	0.0291 (17)	−0.0062 (15)	−0.0118 (14)	−0.0004 (14)
C8	0.0344 (19)	0.051 (2)	0.0214 (17)	−0.0016 (16)	−0.0083 (14)	−0.0062 (15)
C9	0.0267 (17)	0.043 (2)	0.0249 (17)	−0.0097 (14)	−0.0056 (13)	−0.0117 (14)
C10	0.0396 (19)	0.0333 (19)	0.0216 (16)	0.0021 (15)	−0.0027 (14)	−0.0107 (13)
C11	0.0351 (18)	0.0315 (18)	0.0262 (17)	−0.0017 (15)	−0.0039 (14)	−0.0089 (14)
C12	0.051 (2)	0.051 (2)	0.043 (2)	0.0067 (19)	−0.0047 (18)	−0.0211 (19)
N1	0.0312 (15)	0.0335 (15)	0.0240 (14)	−0.0003 (12)	−0.0086 (11)	−0.0059 (11)
N2	0.0368 (15)	0.0339 (15)	0.0209 (14)	0.0019 (12)	−0.0071 (12)	−0.0104 (11)
O1	0.0336 (12)	0.0337 (13)	0.0241 (11)	0.0067 (10)	−0.0051 (9)	−0.0077 (9)
O2	0.0381 (13)	0.0494 (15)	0.0235 (12)	−0.0015 (11)	−0.0046 (10)	−0.0143 (10)
O3	0.0432 (14)	0.0339 (13)	0.0193 (11)	0.0092 (11)	−0.0040 (10)	−0.0068 (9)
O4	0.0522 (16)	0.0382 (15)	0.0287 (13)	0.0167 (12)	−0.0068 (11)	−0.0055 (11)
O5	0.0431 (15)	0.0411 (15)	0.0464 (15)	0.0105 (12)	−0.0076 (12)	−0.0198 (12)
Co1	0.0287 (3)	0.0336 (4)	0.0202 (3)	−0.0038 (3)	−0.0041 (2)	−0.0092 (2)
O6	0.0604 (17)	0.0434 (15)	0.0260 (12)	−0.0160 (13)	−0.0119 (12)	−0.0036 (11)
O7	0.0298 (13)	0.0549 (16)	0.0304 (13)	−0.0001 (11)	−0.0061 (10)	−0.0061 (11)
O8	0.0415 (14)	0.0399 (14)	0.0288 (12)	−0.0091 (11)	−0.0055 (10)	−0.0117 (10)
O9	0.0488 (16)	0.0482 (16)	0.0418 (15)	−0.0069 (13)	−0.0050 (12)	−0.0029 (12)
O10	0.0388 (15)	0.0585 (17)	0.0375 (14)	0.0058 (12)	−0.0061 (11)	−0.0168 (12)
O11	0.0525 (17)	0.0562 (17)	0.0404 (15)	−0.0107 (13)	−0.0101 (13)	−0.0095 (13)

### Geometric parameters (Å, °)

**Table:**

Cu1—O1	1.877 (2)	C10—H10A	0.9700
Cu1—N2	1.887 (3)	C10—H10B	0.9700
Cu1—N1	1.913 (3)	C11—O4	1.229 (4)
Cu1—O3	1.977 (2)	C11—O3	1.278 (4)
C1—O1	1.315 (4)	C12—O5	1.426 (4)
C1—C2	1.406 (4)	C12—H12A	0.9600
C1—C6	1.416 (4)	C12—H12B	0.9600
C2—C3	1.371 (5)	C12—H12C	0.9600
C2—H2	0.9300	Co1—O7	2.075 (2)
C3—O5	1.361 (4)	Co1—O7^i^	2.075 (2)
C3—C4	1.394 (5)	Co1—O8^i^	2.076 (2)
C4—C5	1.364 (5)	Co1—O8	2.076 (2)
C4—H4	0.9300	Co1—O6	2.081 (2)
C5—C6	1.404 (4)	Co1—O6^i^	2.081 (2)
C5—H5	0.9300	O6—H6A	0.8345
C6—C7	1.427 (4)	O6—H6B	0.8434
C7—N1	1.276 (4)	O7—H7A	0.8307
C7—H7	0.9300	O7—H7B	0.8112
C8—N1	1.465 (4)	O8—H8D	0.8503
C8—C9	1.508 (5)	O8—H8C	0.8499
C8—H8A	0.9700	O9—H9A	0.8497
C8—H8B	0.9700	O9—H9B	0.8485
C9—O2	1.258 (4)	O10—H10C	0.8493
C9—N2	1.317 (4)	O10—H10D	0.8488
C10—N2	1.440 (4)	O11—H11A	0.8484
C10—C11	1.516 (4)	O11—H11B	0.8481
			
O1—Cu1—N2	175.78 (11)	O3—C11—C10	117.5 (3)
O1—Cu1—N1	96.59 (10)	O5—C12—H12A	109.5
N2—Cu1—N1	84.11 (11)	O5—C12—H12B	109.5
O1—Cu1—O3	96.05 (9)	H12A—C12—H12B	109.5
N2—Cu1—O3	83.37 (10)	O5—C12—H12C	109.5
N1—Cu1—O3	167.30 (10)	H12A—C12—H12C	109.5
O1—C1—C2	117.3 (3)	H12B—C12—H12C	109.5
O1—C1—C6	124.7 (3)	C7—N1—C8	122.0 (3)
C2—C1—C6	118.1 (3)	C7—N1—Cu1	124.6 (2)
C3—C2—C1	121.5 (3)	C8—N1—Cu1	113.4 (2)
C3—C2—H2	119.2	C9—N2—C10	125.0 (3)
C1—C2—H2	119.2	C9—N2—Cu1	118.7 (2)
O5—C3—C2	124.6 (3)	C10—N2—Cu1	116.28 (19)
O5—C3—C4	114.6 (3)	C1—O1—Cu1	124.7 (2)
C2—C3—C4	120.9 (3)	C11—O3—Cu1	114.35 (19)
C5—C4—C3	118.2 (3)	C3—O5—C12	118.5 (3)
C5—C4—H4	120.9	O7—Co1—O7^i^	179.998 (1)
C3—C4—H4	120.9	O7—Co1—O8^i^	86.87 (9)
C4—C5—C6	123.1 (3)	O7^i^—Co1—O8^i^	93.13 (9)
C4—C5—H5	118.4	O7—Co1—O8	93.13 (9)
C6—C5—H5	118.4	O7^i^—Co1—O8	86.87 (9)
C5—C6—C1	118.2 (3)	O8^i^—Co1—O8	179.999 (1)
C5—C6—C7	117.6 (3)	O7—Co1—O6	89.45 (10)
C1—C6—C7	124.2 (3)	O7^i^—Co1—O6	90.55 (10)
N1—C7—C6	125.2 (3)	O8^i^—Co1—O6	88.61 (9)
N1—C7—H7	117.4	O8—Co1—O6	91.39 (9)
C6—C7—H7	117.4	O7—Co1—O6^i^	90.55 (10)
N1—C8—C9	110.3 (3)	O7^i^—Co1—O6^i^	89.45 (10)
N1—C8—H8A	109.6	O8^i^—Co1—O6^i^	91.39 (9)
C9—C8—H8A	109.6	O8—Co1—O6^i^	88.61 (9)
N1—C8—H8B	109.6	O6—Co1—O6^i^	179.999 (1)
C9—C8—H8B	109.6	Co1—O6—H6A	115.2
H8A—C8—H8B	108.1	Co1—O6—H6B	119.2
O2—C9—N2	126.1 (3)	H6A—O6—H6B	110.8
O2—C9—C8	120.4 (3)	Co1—O7—H7A	115.5
N2—C9—C8	113.5 (3)	Co1—O7—H7B	119.8
N2—C10—C11	108.2 (3)	H7A—O7—H7B	114.9
N2—C10—H10A	110.1	Co1—O8—H8D	109.1
C11—C10—H10A	110.1	Co1—O8—H8C	109.7
N2—C10—H10B	110.1	H8D—O8—H8C	109.4
C11—C10—H10B	110.1	H9A—O9—H9B	109.9
H10A—C10—H10B	108.4	H10C—O10—H10D	109.7
O4—C11—O3	123.8 (3)	H11A—O11—H11B	110.0
O4—C11—C10	118.7 (3)		

Symmetry codes: (i) −*x*+2, −*y*+1, −*z*+1.

### Hydrogen-bond geometry (Å, °)

**Table:**

*D*—H···*A*	*D*—H	H···*A*	*D*···*A*	*D*—H···*A*
O6—H6A···O11	0.83	1.94	2.771 (4)	174
O6—H6B···O3^ii^	0.84	1.93	2.765 (3)	169
O7—H7A···O9^iii^	0.83	1.95	2.757 (3)	165
O7—H7B···O10	0.81	1.94	2.723 (3)	162
O8—H8C···O2^iv^	0.85	2.31	2.812 (3)	118
O9—H9A···O10	0.85	1.99	2.769 (4)	152
O9—H9B···O1	0.85	1.96	2.798 (3)	172
O10—H10C···O2^v^	0.85	2.02	2.783 (3)	149
O10—H10D···O4^ii^	0.85	2.00	2.844 (4)	173
O11—H11A···O2^vi^	0.85	2.09	2.916 (3)	164
O11—H11B···O9	0.85	2.00	2.844 (4)	176

Symmetry codes: (ii) *x*+1, *y*, *z*; (iii) −*x*+1, −*y*+1, −*z*+1; (iv) −*x*+1, −*y*+1, −*z*+2; (v) −*x*, −*y*+1, −*z*+2; (vi) *x*+1, *y*, *z*−1.
